# The understanding of healthcare workers on the content of palliative care policy in Shesilweni Swaziland: a qualitative study

**DOI:** 10.3332/ecancer.2018.857

**Published:** 2018-08-10

**Authors:** Teluleko Nhlonipho Maseko, Collin Pfaff, Aziza Mwisongo

**Affiliations:** 1 Department of Rural Health, School of Public Health, University of the Witwatersrand Johannesburg, Johannesburg 2193, South Africa; 2 Department of International Health Program, National Yang Ming University, Taipei City 11221, Taiwan; 3 Department of Rural Health, School of Public Health, University of the Witwatersrand Johannesburg, Johannesburg 2193, South Africa; 4 Centre of Health Policy, School of Public Health, University of the Witwatersrand Johannesburg, Johannesburg 2193, South Africa

**Keywords:** palliative care, policy, Africa and knowledge

## Abstract

We undertook a qualitative study design to explore the understanding of healthcare workers of the content of the palliative care policy in Swaziland. A total of 17 health workers participated in this study. The study showed that there was a lack of knowledge about palliative care, confusion as to where palliative care should be offered and by whom and the role of providing medication as a part of palliative care. Interestingly, the health workers mentioned the importance of different actors, the importance of teamwork and some perceived successes of palliative care implementation. Several challenges were reflected which included the availability of medicines, human resources, transport, infrastructure and a lack of coordination of Rural Health Motivators. Suggested strategies for improving palliative care which have been pointed out include training, improving medicine supply and organisational issues.

## Palliative care policy in Africa

In Africa, three of 54 countries (Mozambique, Rwanda and Swaziland) have established a stand-alone national implementation plan for palliative care services [2], whereas seven countries (the Democratic Republic of Congo, Kenya, Mauritius, Namibia, South Africa, Tanzania and Uganda) have integrated palliative care into public healthcare policy or into a strategic plan focusing on cancer treatment [26]. Malawi has published national palliative care guidelines, whereas other countries, including Botswana, Ethiopia, Nigeria, Zambia and Zimbabwe, are drafting their national palliative care policies [12].

The standalone implementation plan in the three countries has seen the palliative care program viewed as an important service on its own, whereas integrated palliative care services are viewed as a complimentary service to HIV/AIDS. In addition, the standalone plan encompasses a broad spectrum of diseases. The challenge of both policies is monitoring the implementation of them.

In Swaziland, the palliative care policy was developed in 2011 and both documents palliative care and considers palliative care as a human right [46]. Swaziland currently has no national strategies for cancer or other non-communicable diseases but does have a strategy for home-based care that includes palliative care [42]. The country has a dedicated budget line for: palliative care in its health budget, a palliative care desk and a dedicated palliative care officer in the Ministry of Health (MOH).

## Swaziland national palliative care policy

The gaps identified in the 2009 review of palliative care services led to the development of a national palliative care policy in 2011. The national palliative care policy was the product of widespread consultations and participation of institutions and individuals. The policy provided a broad guide for the implementation and management of palliative care interventions, programs and activities at various levels [43]. The policy was supported and launched by the Minister of Health and had a lot of public support [43].

The purpose of the policy was to provide a framework for standardised implementation of palliative care services in Swaziland. This is because palliative care is a broad concept encompassing a range of activities undertaken by health workers. Furthermore, the policy was a tool to ensure good management of patients needing palliative care, guide priority strategic orientations in relation to palliative care and secure resources (human, material and financial) [43]. Our research question is; what is the understanding of healthcare workers on the content of palliative care policy?

## Methods

### Study design

A qualitative exploratory design using in-depth interviews was used. A qualitative method was used because in-depth information was required on the implementation of the policy, for instance, information about the challenges and successes in the implementation of the palliative care policy. The depth of the information gained was likely to be greater than with a questionnaire and also the interview was an opportunity to explore ambiguous responses. Such information is expected to enable policymakers, partners and MOH palliative care coordinators to better address the policy implementation gap.

- Inclusion criteria: Diploma holders in medical training, working with patients with life-limiting illness and with a minimum of 3 years of experience.
- Exclusion criteria: Administrative personnel, no diploma and lay support stuff such as orderlies.

### Study site

The study was conducted in the South Western Shiselweni Region which is a lowveld ecological region that is furthest away from the capital Mbabane, and adjoins the KwaZulu–Natal Province of South Africa to the south. It was selected because it is the most rural district in Swaziland. It has an area of 3,786.71 km^2^ and a population of 208,454, and is divided into 14 tinkhundla, its administrative centre being Nhlangano (population census, 2007). The region has one referral hospital, two health centres, 17 clinics and several non-governmental organizations (NGOs) providing health services [41].

### Study population and sample

The population for this study involved healthcare workers in Shiselweni Region, from five facilities; one regional hospital (Hlatikulu), health centre (Nhlangano) and three rural clinics which were purposefully selected by the researcher.

Purposive sampling was used, as the researcher wanted to ensure the sample drew on participants who are informed and involved with the routine implementation of palliative care. A total of four healthcare workers from each facility were supposed to be interviewed, which would have resulted in 20 respondents. Facilities were purposively sampled in order to ensure that the sample covered a full range of possible characteristics of interest these included providing anti-retroviral therapy services and remoteness of clinic. The following health workers were interviewed: chief medical officer, matron, doctor, pharmacist, physiotherapist or sister in-charge and registered nurse The researcher only managed to recruit 17 respondents because they were engaged in their daily duties and could not participate in the study.

## Data analysis

Verbatim transcripts of the interviews were analysed using the student version MAX QDA software. The transcriptions were de-identified and participant’s names were substituted by codes for confidentiality. The transcripts were then exported to MAX QDA software for the qualitative data analysis. Subsequently, the researcher familiarised himself with the transcripts by reading and re-reading them to enable a clear understanding of the data and to facilitate the development of codes. The coding process was based on key themes which were constructs that were identified before data collection from the literature review and from the experience of the researcher. For quality control in coding, an inter-coder agreement was achieved through the supervisor reading transcripts that had been coded by the researcher. Through the inter-coding reliability process, the definitions were refined as needed for better clarity.

## Ethical considerations

The study was granted ethical clearance by the Human Research Ethics Committee of the University of Witwatersrand in South Africa (Certificate No: M 141039) and also the Human Scientific Ethics of Swaziland under the MOH in Swaziland (REF:MH/599C/ FWA 00015267/ IRB 0009688). All interviews were conducted in English.

## Results

A total of 20 potential interviewees were approached. However, it was only possible to interview 17 respondents: clinical workload for some participants was too great to enable participation in the study.

Only 60% of the study population was aware of the palliative care policy. The participants (both females and males) were from a range of nationalities, most of them used Siswati and Shona as a medium of communication in their homes. Table 1 shows the demographics of the study population.

All respondents were grateful to be part of this study and their responses are presented below in different themes.

## Knowledge about palliative care

In general, most of the health workers, including the policymakers, understood the concept of palliative care. They were aware of the scope of work with respect to palliative care and of the available guidelines that were designed to assist with providing standards and norms for managing life limiting and threatening conditions.

‘Palliative care is taking care of those with long illnesses so that we provide them with the support they need until the die’ (Nurse, Health Centre, female).

Most health workers were aware that there was a policy with regards to palliative care but the content of the policy was not clear to them. Some did not know that a policy existed at all. Others felt that the policy was a good document, and wanted to know how it could best be implemented.

‘I am aware but I have not seen it. I know that there is a policy for that’ (Nurse, rural clinic, female).

‘Policies are documents given to the matron and they are kept in their offices, I personally don’t know any palliative care policy’ (Nurse, rural clinic female).

‘I personally feel the policy is okay, I think we need the necessary equipment to implement it. Otherwise, it is a good policy which we can be implemented. It can be of good use to the people out there’ (Nurse, rural clinic 2, female).

Some healthcare workers specifically expressed a lack of knowledge about the concept of palliative care and said that this was common to the health workers in rural clinics whose primary role was to provide primary healthcare.

‘I am blank on palliative care. I have minimal knowledge about it. I can’t really say much about it, unlike the other services we offer’ (Nurse, rural clinic, female).

‘I am yet to attend that policy workshop, because right now I am still blank on how it’s functioning’ (Nurse, health centre, female).

One respondent believed that some training in the policy would assist her in understanding how to implement it and to know what is expected from her, saying:

‘Unfortunately no, I am not aware. I have not attended any workshop or any in-service training on palliative care and it is not something that is taken so seriously’ (Nurse, rural clinic, female).

Thus, it seems that although the general concept of palliative care was understood, there were significant gaps in knowledge of the content of the policy and knowledge about providing palliative care.

## Confusion as to where palliative care should be offered and by whom

The interviewed health workers and policymakers presented different views about the level of services where palliative care should be offered. The policymaker believed that palliative care was mainly offered in hospitals as it was a service offered to patients who had cancer. Respondents who worked at health centres had different descriptions from this, describing palliative care as offering services to patients at home.

‘It is actually a holistic approach, I think it is offered to a dying patient or a cancer patient, whatever the case may be, but you actually find that being in the hospital setting with clinicians’ (National Policymaker, MOH, female).

‘I think it is taking care of sick people at home, not at the hospital or long-term illness like cancer, something that is not curable’ (Nurse, regional referral hospital, male).

‘It is giving care to people who are about to die, where we want them to die peacefully regardless of where they are. Palliative care can be offered at home and hospitals’.

The policy itself though clearly stated that palliative care should be offered at all levels. This concept was understood by some respondents. For example, one health worker wanted more sensitisation for the community to be aware that palliative care was available at every level.

‘What I understand about the policy, is that, they are trying to decentralise the palliative care to all areas of the country, especially the peripheries, where we were having some of the clients going to a central point to collect their medication and get the care they need’ (Senior Matron, Head of facility, female).

‘We are achieving but I think there is a lot still needed to be done especially in terms of sensitising some health workers and the community at large to be aware that they can access medication for palliative care at the nearest health facility should they be prescribed for them’ (Nurse, rural clinic, female).

It was also not clear to some respondents as to who should be offering palliative care services. Nurses working in the rural clinics perceived palliative care as being offered by NGOs and mainly in urban areas.

‘I think palliative care is offered by NGOs and in urban areas not in rural clinics’ (Nurse, rural clinic, female).

‘In my opinion, the palliative care policy was developed for NGOs to implement it, not us’ (Nurse, rural clinic female).

## The role of providing medication as part of palliative care

There was a variety of opinion in regards to how medical or holistic palliative care was viewed. Many respondents mentioned the important role of providing medication. This also included counselling on how to take the medication. The importance of morphine was specifically mentioned including the advantage of various preparations. The importance of preventing stock outs was mentioned. The fact that morphine was not available at health centres and patients had to be referred for this was also mentioned.

‘So in my job, I have to make sure that I explain and discuss with every patient as to why they are taking that medication, how that medication is going to help them and the importance of that medication’ (Senior Matron, health centre, female).

‘I can speak for myself, as a pharmacist, I have to make sure that the drugs they are using are always in stock and make sure that they are given in the right dosages, and so that they are not toxic to the patient’ (Pharmacist, regional hospital, female).

‘We usually provide panado only. We send them to the hospital for physiotherapy and others’ (Nurse, rural clinic, female).

‘Tablets are rather heavy while the syrup seems a little bit lighter and does not make them too drowsy. I’m not sure but that’s my perception. The patient must not be flat out but have his or her pain controlled’ (Senior Matron, health centre, female).

However, it was not only medication which was needed but also other medical supplies as well.

‘I am also talking about providing material resources such as diapers, artificial dressings that you discard. So our role is to see that all these things are available’ (Senior Matron, Health centre, female).

Other respondents had a more holistic view of palliative care services beyond medication and supplies. They mentioned the role of counselling and also of other team members.

‘In terms of what as each role is very important, nurses will do their nursing part, including monitoring adherence to taking medications, giving injections, the physiotherapist will give physical treatment through non-pharmacological means’ (Physiotherapist, regional hospital, male).

‘I give patients medicine, I also provide counselling’ (Nurse, rural clinic, female).

‘In as much as a lot of drugs are used to manage patient’s pain, we believe that their pain can also be managed through physical therapy. Therefore, we try to assure that physiotherapy is accessible to the chronically ill patients’ (Physiotherapist, regional hospital, male).

‘I feel like it is one part of healthcare provision that is really lacking especially for I am a trained community nurse. That is one part that is lacking. It is like now we are focusing more on the curative, we are leaving behind rehabilitation because palliative care is the rehabilitation part of healthcare’ (Nurse, rural clinic 1, female)

## Discussion

### Knowledge about palliative care

In Swaziland, a palliative care policy and strategy were developed in 2011 in which palliative care was considered a human right [42]. The development of this policy had a number of factors in its favour: palliative care in Swaziland had a long history and the policy enjoyed the public support of influential policy actors including the health minister. Since that time there has been a dedicated budget line for palliative care in the health budget, a palliative care desk and dedicated palliative care officer in the MOH. According to the results of this study, the policy did translate into increased levels of palliative care provision, although it did not produce greater geographic equity.

The study findings show policymakers had a clear understanding about the policy. However, palliative care was generally more understood by health workers from healthcare centres compared to rural healthcare workers in rural clinics. They were aware of their scope of work with respect to palliative care and of the available guidelines that were designed to assist with providing standards and norms for managing life limiting and threatening conditions. The health workers did, however, present a view that they were unclear about the kind of services they should render under palliative care.

In addition, the study findings do offer a concrete baseline in terms of awareness in regard to palliative care; nevertheless, it neither reflects an increase nor decrease of palliative care services. On the other hand, it is worth noting that the findings provide a clear hint on how the policy could be effectively operationalised. In addition, certain outcomes can be measured quantitatively, thus giving clear direction on how palliative care services can be strengthened in the Shesilweni region.

Some of the findings from this study concur with findings from other published studies. For example, in the North Eastern United States, health workers expressed varying perceptions about the meaning of palliative care. There was confusion regarding the differences between palliative and hospice care, the timing of referral to palliative care was a key issue, and there was the need for further education in palliative care [28]. In the results section, such a phenomenon is reflected which shows that common problems in the provision of palliative care services are similar in some settings. Alarmingly, the physician’s perspective saw palliative care as a supportive service to be given to patients that have been diagnosed with a few months remaining to live. However, other health workers tended to believe that it should be available to all patients, not just as an option after disease-oriented care fails. Furthermore, nurses emphasised the role of palliative care in facilitating discussion and decision making about goals of care and quality of life; issues that they felt other treating physicians frequently neglected [31]. Most rural health workers from the findings reflected that if they could attend a workshop in palliative care they could facilitate such engagement which is often neglected by treating physicians. Others saw palliative care as a signal that healthcare workers had abandoned all hope for a specific patient. A number of participants viewed palliative care as incompatible with the hospital goals of saving lives, and some surgeons perceived instituting palliative care as being ‘soft’ or ‘giving up’ [32]. In this study, similar statements are seen in the results section whereby some respondents believe the policy was made for NGOs and the palliative care was to be offered by NGOs in the urban areas.

The Swaziland National Palliative Care Policy promotes a holistic approach to patient care and outlines proposals for integrating palliative care into Swaziland’s healthcare system. The policy has decentralisation of palliative care as a specific objective and clearly outlines the services that are to be available at three levels—community and clinic level; health centre level and hospital level. However, health workers seemed not to understand or fully implement this because of lack of resources and other working materials at the lowest levels of care.

In addition, health workers understood palliative care differently at different levels thus causing confusion at all levels of care (Swaziland National Palliative Care Policy, 2011).

In many African countries, palliative care has been implemented using a combination of models at many levels in the healthcare service. These countries include Uganda and Kenya, who were the pioneers of palliative care through the African Palliative Care Association. The following models have been explored:

- Hospital-based palliative care teams: These provide palliative care services within a hospital context and can offer generalist, intermediate or specialist functions. This may include both in-patient and out-patient services.
- Home-based care: This is frequently provided through specialist palliative care teams that visit patients and support them directly in the home, or generalist or intermediate care delivered through home-based care services provided by community-based programmes. These may utilise trained volunteers and may have a clear partnership with specialist palliative care services.
- Outreach services: Some palliative care providers have outreach services that support other organisations to provide palliative care, or provide roadside and mobile clinics. In Uganda, outreach services are used in cases, whereby a team of different health workers do outreach services to those who need palliative care services. In most cases are referred by the hospitals that have discharged them.

This study shows that implementing palliative care services is a complex task particularly for rural clinics, especially because certain policies prevented opioid medication to be kept on site. The unavailability of morphine in rural clinics was perceived to have affected the decentralisation of palliative care services.

The issue of regulations around morphine storage and use has been a barrier in many African countries, to decentralising palliative care services at a community or primary healthcare level. Uganda has led the way in this area and was the first African country to adopt the World Health Organisation pain management guidelines and to develop model regulation guidelines for the authorisation, documentation, storage and distribution of morphine. Until recently, opioid prescription in Uganda was limited to registered physicians, dentists and veterinary surgeons under the National Drug Policy and Authority Statute of 1993. This was amend mended in 2004 to allow specialised health workers to prescribe morphine after successful completion of a 9-month training course [19]. This amendment increased accessibility to morphine to a large population of the community, especially in the rural areas.

### The role of providing medication as part of palliative care

While there are some studies of how patients view palliative care services, few studies have explored how physicians and other healthcare providers in acute care hospitals perceive and utilise these services [15].

In a study conducted in Pennsylvania hospitals, most participants perceived palliative care to be a type of care that focuses on terminal pain and symptom management and on facilitating decisions to stop life-sustaining treatments. Some viewed palliative care as reserved for patients with terminal cancer, and some viewed it as care for ‘actively dying’ patients (i.e., during the last days or hours of life) [32].

However, palliative care is ‘an approach that improves the quality of life of patients and their families facing problems associated with life-threatening illness, through the prevention and relief of suffering, the early identification and impeccable assessment and treatment of pain and other problems, physical, psychosocial and spiritual’ [46]. While the palliative care approach was developed in response to the plight of patients with cancer, other life-threatening conditions, such as HIV, heart diseases, diabetics and stroke, were soon added. The focus is therefore on life threatening illnesses that are malignant and non-malignant [2].

Palliative care is thus not only about providing medications as most health workers perceive it to be but is a holistic approach that goes beyond physical aspects only. This is evident in that different health workers who form part of the palliative care team that offers palliative care. One such example in this study was the physiotherapist who used non-pharmacological interventions when dealing patients with life limiting conditions.

### Study limitations

The study was done in one region of the country. More than two-thirds of the respondents were not Swazi nationals and the interviews were conducted in English. English is not the home language of any participants. Another limitation is the short length of service of the respondents. Finally, we acknowledge reflexivity because we were known to the teams, which was a strength for this study.

## Conclusion

Palliative care is a major but often still neglected public health issue requiring national and international policy responses. The findings in this study have indicated a range of issues that contributed to implementing the palliative care policy among health workers in the Shiselweni Region of Swaziland. The issues included a lack of understanding of the role of palliative care, its impact on the rural clinics, and the challenges in human resources. In addition, there is great variation globally in providing palliative care for individuals with life-limiting conditions which needs effective planning and implementation of programs and policies, hence improving international mortality and morbidity rates.

### Recommendations

This study would suggest that training and mentorship programs should be implemented for the success of managing cancer cases in health facilities. The government of Swaziland should effectively operationalise the palliative care policy in collaborating with key stakeholders like Non-Governmental Organizations who have been established in the palliative care fraternity in the past more especially those organisations that offered palliative care to patients with cancer.

## Conflicts of interest

The authors declare that there are no competing interests.

## Funding

There was no funding for this research.

## Figures and Tables

**Table 1.** Demographics of study population.

Occupation	Number of participants	Level of facility	Nationality	M/F	Years of service	Age range
Policymaker	3	National	Swazi	F	3	35–45
		Stakeholder	Swazi	M	4	40–45
Chief Medical Office (head of facility)	1	Regional hospital	Non-Swazi	M		40–50
Doctor	1	Health centre	Non-Swazi	F	5	25–35
Matron (Head of facility)	1	Health centre	Swazi	F	6	45–55
Nurses	8	Rural clinic 1	1 Swazi 1 non Swazi	F	4	35–45
		Rural clinic 2	3 Non Swazi 2 Swazi	F	3	35–45
		Health centre	1 Swazi	F	3	35–45
Physiotherapist	1	Regional hospital	Non-Swazi	M	3	45–55
Pharmacist	1	Health centre	Non-Swazi	M	4	35–40
Pharmacist	1	Regional hospital	Swazi	M	4	30–35
